# Use of a Health Advocacy Model for Survivors of Interpersonal Violence

**DOI:** 10.3390/ijerph17238966

**Published:** 2020-12-02

**Authors:** Jessica Grace, Scott T. Walters, Irene Gallegos, Erika L. Thompson, Emily E. Spence

**Affiliations:** 1 Department of Public Health Education, School of Public Health, University of North Texas Health Science Center, Fort Worth, TX 76107, USA; emily.spence-almaguer@unthsc.edu; 2 Department of Health Behavior and Health Systems, School of Public Health, University of North Texas Health Science Center, Fort Worth, TX 76107, USA; scott.walters@unthsc.edu (S.T.W.); erika.thompson@unthsc.edu (E.L.T.); 3 Department of Kinesiology, University of Texas Arlington, Arlington, TX 76109, USA; irene.gallegos@uta.edu

**Keywords:** intimate partner violence, health advocacy, women’s health, motivational interviewing, solution-focused therapy, goal attainment scaling

## Abstract

This article examines the implementation of a health advocacy model designed for survivors of interpersonal violence (IPV) in a metropolitan area of North Texas. Using a framework influenced by motivational interviewing, solution-focused therapy, and trauma-informed care, this program engaged IPV survivors in creating health and safety goals. Goal attainment scaling was used to track progress after each health advocacy encounter. Clients could set their own goals for healthcare, self-care, and safety. The program served 419 clients and 648 goals were set by clients at the first visit. Among all goals, 89% selected goals focused on healthcare, with 47% of those selecting obtaining health insurance or coverage as a need. These results demonstrate the need for an enhanced healthcare response for this population. The remaining goals selected were self-care (7%) and safety (3%). The design of the health advocacy intervention shows promise towards filling the gaps between IPV and healthcare service delivery systems.

## 1. Introduction

### 1.1. Interpersonal Violence

Interpersonal violence (IPV) is a public health crisis that intersects multiple disciplines, including social services, healthcare, and criminal justice. Interpersonal violence can be verbal, physical, sexual, or any other form of abuse against another person or group of people [1,2]. In a recent survey, more than one in three women (36.4% or 43.6 million) in the US reported experiencing intimate partner violence, a type of IPV involving a current or former intimate partner, during their lifetime, and about one in four women (25.1% or 30.0 million) reported an intimate partner violence-related impact (e.g., health or social consequence; [3]). Rates were similar for men who experienced intimate partner violence in their lifetime (33.6% or 37.3 million); however, fewer men reported an intimate partner violence-related impact (10.9% or 12.1 million; [3]).

IPV can produce a range of acute and chronic health consequences. For instance, individuals who experience IPV have an increased likelihood of gynecological, digestive, cardiovascular, central nervous system, chronic stress, and psychological health issues [4,5,6]. These health consequences lead to healthcare utilization rates and costs that are higher for victims than non-victims [7,8,9]. Furthermore, since IPV crosses many sectors, such as criminal justice, social services, and education, the costs tend to be multiplied. The total economic cost of IPV in the U.S. is estimated at $3.6 trillion based on 43 million adults with any lifetime victimization and a lifetime cost of $103,767 per female victim and $23,414 per male victim [10].

For this reason, a multi-systems level approach is necessary to address IPV. Identifying the needs of victims and coordinating care requires a bridge between IPV responses in community settings, such as social services and the healthcare community. In that effort, health advocacy offers a linkage by embedding advocates in both settings to coordinate healthcare services while at the same time providing broader wellness coaching and crisis support.

### 1.2. Health Advocacy

Health advocacy is a framework that facilitates information sharing and resource referrals to individuals to increase patient autonomy and reduce morbidity or mortality [11]. In the broader healthcare system, health advocates often perform health navigation activities, including ensuring access to care, mobilizing resources, addressing health inequities, influencing health policy, and creating system change [12]. In contrast, health advocacy stationed in the environment of an IPV setting is a novel approach that prioritizes the health needs of survivors as critical to their overall success. Health advocates are skilled in navigating healthcare resources, solution-focused dialogues, and other client-centered strategies that promote health, safety, and stress reduction. Advocates work alongside case managers and counselors in a holistic approach that includes the mental and physical health needs of individuals in addition to their tangible needs, such as housing, childcare, and income.

This paper describes the health advocacy process that was developed to identify and address IPV within healthcare and social service settings. The Technology Enhanced Screening and Supportive Assistance (TESSA) program, which had health advocacy as a key component, had three theoretical influences for health advocacy (Table 1): motivational interviewing, solution-focused brief therapy, and trauma-informed care.

Motivational interviewing (MI) is a client-centered counseling strategy for strengthening a client’s motivation and commitment to change [13]. MI is an evidence-based approach that has been used in many settings, including for victims of intimate partner violence [14]. Likewise, solution-focused brief therapy (SFBT) is a directive approach that helps clients translate skills and strengths into future goals [15]. Solution-focused approaches help clients to identify skills that will best help them make incremental progress toward their goals [16]. A major component of the TESSA health advocacy program was client goal setting for their health, safety, and self-care needs. SFBT and MI share many overlapping strategies: both are non-judgmental, future-focused, and emphasize client autonomy. Moreover, there is evidence that the two styles can play complementary roles in behavior-change interventions [17]. MI’s focus on autonomy support, evocation, and collaborative decision-making is complemented by SFBT’s promotion of patient expertise, solution-focused talk, and resourcefulness. The two work together to create a client who is empowered and has a voice in the change process. Finally, trauma-informed care (TIC) is an evidence-based approach that recognizes the impact of past trauma on current health and patient care experience [18]. TIC focuses on engaging clients in a collaborative, non-judgmental style with an appreciation for the way trauma influences health behavior [19].

The TESSA health advocacy approach combines health coaching (i.e., setting wellness and health goals), health navigation (i.e., linkages to health services and insurance), and crisis intervention (i.e., safety planning) into an innovative and integrative model. The intervention frameworks MI, SFBT, and TIC were used to help participants set individualized health and safety goals. The purpose of this paper is to describe the implementation of the TESSA health advocacy approach, including the types of goals set by the IPV clients who participated in the program. By reviewing the types of goals selected by clients who experienced IPV, we can understand the broad range of needs among these clients and the potential for a holistic perspective of health, wellness, and safety. Moreover, the design of a health advocacy program for IPV organizations and healthcare may serve as a template for other populations with complex needs.

## 2. Materials and Methods

### 2.1. Setting

The setting for this project was a metropolitan county in North Texas. As a community demonstration project funded by the Office on Women’s Health, TESSA’s community partners included three medical systems (one university-based, one county hospital system, and one federally qualified community health center), along with three local IPV provider agencies (one domestic violence shelter, one rape crisis center, and one family justice center). The program worked with individual clinics in each health system and IPV agencies to identify people with a current or past history of IPV. Patients and clients with a history of IPV were then referred to health advocates using multiple methods (e.g., referrals from IPV organizations and clinic-based screening for IPV). Health advocates were responsible for engaging clients in service delivery and coordinating care between IPV organizations (the rape crisis center, domestic violence emergency shelters, and family justice center) and primary care clinics. Health advocacy was implemented with an expanded scope to guide clients in achieving health, wellness, and safety goals. This paper reports data from 419 people enrolled into the project between 1 September 2016 and 28 February 2019.

### 2.2. Ethical Considerations

The health advocacy program was delivered to clients participating in the TESSA program. Participation in TESSA health advocacy services were voluntary and offered to any individual that screened positive for IPV, whether in a clinic or IPV agency. The analysis of these deidentified data were approved by the North Texas Regional Institutional Review Board.

### 2.3. Training Health Advocates

Health advocacy requires a unique set of skills in order to address the medical, behavioral, and safety concerns that may arise in interactions with people who have experienced IPV. TESSA health advocates were trained in motivational interviewing, solution-focused approaches, and trauma-informed care, which formed the foundation of the intervention. Health advocates also received training in specific IPV topics, such as protective orders, strangulation, healthcare assistance programs, homelessness, and safety planning. Health advocates were not intended to be experts in each of these areas; rather, it was intended that they possess a sufficient understanding of health, wellness, and safety to support clients in achieving their specific goals.

### 2.4. Health Advocacy Delivery

Upon initial contact with a client, health advocates would utilize motivational interviewing and solution-focused questions to help clients identify their needs and reflect on strategies that may help them address those needs. As part of the intake, clients were presented with an icon-based worksheet with 24 images related to healthcare management, self-care, and safety. Clients were asked to reflect on which of these 24 areas were of highest priority. They would then select each goal area and rate it on a scale from 1 to 10 (1 = “Not a big priority right now” and 10 = “This is an area that I want to work on right away”). Those initial priority ratings served as the baseline for services and were re-evaluated at each encounter with a health advocate. This sheet established the client’s priorities and emphasized the mutuality of the advocate/client relationship. In addition, the approach to safety planning was intended to be more client-directed, using open-ended questions and icons to elicit perceived risks, in contrast to traditional safety plans that include standardized sections and required responses. Health advocates start by asking clients what strategies have been successful in the past, encouraging them to continue to use those strategies, alongside other methods that may be generated during the sessions. A final component of health advocacy that is unique to this project is the blending of health coaching and self-care. As trauma impacts the body, it creates added stress and tension. When advocates talked with clients about self-care, they explored methods of self-regulation, such as mindfulness, meditation, yoga, physical exercise, nutrition, and other practices that clients may use to deal with stress.

### 2.5. Measures

Goal attainment scaling was used to evaluate the health advocacy intervention on client goal selection for each of the 24 priority goal domains. Goal attainment scaling is a method of evaluation that uses individually identified items and scales to monitor incremental changes in client outcomes over time [20]. Originally developed in 1968 for mental health treatment, goal attainment scaling has since been effectively used in multiple settings, including rehabilitation centers and geriatric services [21].

The goal attainment method uses a scale ranging from −2 to +2 to identify the likely or desired treatment outcomes [20]. This scale spans from −2, which represents the most unfavorable outcome, to a 0 being the most likely or expected outcome, and +2 the most favorable outcome. For the 24 goals included on the TESSA icon worksheet, the development team established operational definitions for each level of attainment/status from −2 to +2. Health advocates would dialogue with a client to determine what level of baseline goal attainment aligned with that client’s circumstance when the goal was established. In this respect, while each level of the goal was standardized, the baseline score for each client would vary based on that individual’s situation. For example, a goal to obtain a primary care provider might range from −2 (use the emergency room for primary care) to +2 (secure a primary care physician and attend at least one appointment). Likewise, a goal to increase safety in the home might range from −2 (intentionally attempting to escalate conflict/risk at home) to +2 (implementing steps to increase safety at home and identifying one observable benefit). Goal attainment scores were attached to every goal; multiple goal attainment scores were entered for the same person if there were multiple goals. This study includes an analysis of the baseline goals selected by clients receiving health advocacy services.

### 2.6. Data Analysis

Descriptive frequencies were estimated for all demographic characteristics and referral location where available. Providing demographic characteristics was not mandatory for clients to engage in health advocacy services and resulted in unreported demographic data for some clients. The number of clients setting a goal and average goal attainment score was estimated for each goal category and goal, and an overall average goal attainment score was also estimated.

## 3. Results

Between September 2017 and February 2019, the health advocates interacted with 419 clients. Approximately 80% clients were female (Table 2). The average client age was 36.9 years with a range of 19 to 72 years. Almost one-third of the clients identified as being Hispanic and one-third as White. Most clients were referred from an IPV organization.

**Figure 1:**
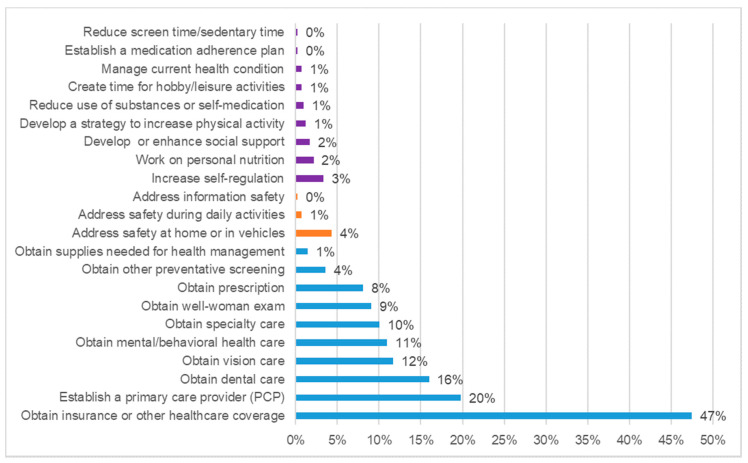
Proportion of health advocacy clients with selected goals by goal type (*n* = 419).

During the clients’ first encounters with the health advocates, the average number of goals set were 1.87 (range: 1 to 9 goals). At total of 648 goals were set by 419 clients at the first visit (Figure 1). Approximately 9 out of 10 (89%) goals set were within the healthcare domain, with obtaining insurance or other healthcare coverage as the main goal in this category (47% of those clients selecting goals in this area). Other common goals in the healthcare domain included establishing a primary care provider (20%), obtaining dental care (16%), obtaining vision care (12%), obtaining mental/behavioral health care (11%), obtaining specialty care (10%), obtaining well-women’s exams (9%), and obtaining prescriptions (8%). At the time of goal setting, the majority of goal attainment scores fell into the −1 to 0 range (Table 3), indicating that clients either had not identified a provider or identified a provider but had not yet made an appointment. Within the healthcare domain, obtaining a well-women’s exam was the lowest rated goal with an average of −1, representing no identified provider had been selected. Obtaining supplies needed for health management had an average of 0 rating for 6 clients (the highest average in the healthcare domain), indicating the client had identified resources for help in obtaining the needed supplies/equipment. Self-care was the second most common goal domain (7% of clients selected this domain). The most common goals in the self-care domain included increasing self-regulation (3%) with an average goal attainment score of −0.29, representing clients either not identifying methods for controlling physiological functions or identifying methods, but not yet implementing them. Among all the goals selected, 3% were related to safety, which included 4% of clients reporting a goal to address safety at home or in vehicles. The average safety-related goal attainment baseline value was close to 0 (−0.11), indicating clients had identified methods to increase safety at home but had not yet implemented them. 

## 4. Discussion and Conclusions

This paper describes the initial implementation of a health advocacy program for victims of interpersonal violence and the self-selected goals set by clients in this program. Based on the client reported healthcare, safety, and self-care goals from the utilization of the goal attainment scale, it supports the need for holistic healthcare linkages among this population. Specifically, the majority of goals set by TESSA clients were focused on healthcare, emphasizing the health-related needs of this population of persons who have experienced IPV. The utilization of motivational interviewing, solution-focused therapy, and trauma-informed care in the health advocacy model, encouraged survivor autonomy to select goals relevant to their immediate needs that may not be currently met by other services. For providers and programs serving victims of IPV, these results indicate an opportunity for integration of healthcare services into more traditional social service settings. Increased collaboration with local healthcare providers to provide onsite navigation or linkage to victims could address these needs in settings where specialized health advocates would not be feasible.

An effective community-level response to interpersonal violence requires a cross-system coordination of crisis intervention, housing, criminal justice, mental and physical health resources, legal services, childcare, and other supports. The service utilization patterns in this project highlight the critical importance of being able to access health and mental health care following victimization, as demonstrated by the goals set by the clients in this study [22]. While studies of healthcare use by victims of intimate partner violence show higher costs and utilization than non-victims [7,8,9], the most commonly selected goal of obtaining healthcare insurance or other coverage reinforces the importance of accessing healthcare as a basic need. In a systematic review of research on healthcare practitioners’ responses to domestic violence and abuse [23], settings that featured team collaboration across disciplines reported the highest levels of support. The integrative TESSA model has the potential to facilitate cross-sector collaboration through its emphasis on bi-directional navigation in IPV and health systems of care.

Motivational interviewing (MI) and solution-focused therapy (SFT) share many similarities in application: empathic listening, highlighting clients’ strengths and resources, focusing on what is working versus what is not working, and negotiating measurable goals [13,24,25]. In these interactions, collaboration plays a key role in empowering clients. In a collaborative conversation, the client and provider are working together to solve a problem; clinicians who are high in collaboration are curious about the client’s perspective and are willing to be influenced by the client’s ideas and experiences, recognizing that clients bring their own expertise to the interaction. Clinicians use their professional expertise strategically, and like never before the client is ready to receive it. The intentional focus on collaboration promotes shared decision-making, helping the client to take ownership of any goals that are set during the interaction. Providing IPV advocates and clinicians, trained in these empowerment-focused approaches, creates the potential to improve the response to survivors in communities where resources are limited. Blending MI and SFT has demonstrated positive results in a variety of clinical and medical settings [17,26], complements a trauma-informed care mindset, and offers a unique framework for survivors of interpersonal violence. An icon-based intake worksheet and customized safety plan further emphasized the collaborative style of engagement between advocate and client, and the trauma-informed principles of promoting choice and shared control. Standardizing metrics for each goal attainment scale prior to implementation allowed the team to assess client progress at each encounter, demonstrating incremental change regardless of service duration. It also provides data necessary for program evaluation and research.

## 5. Limitations

This study has a number of limitations. First, the study was designed to be a service-delivery project with an evaluation component, rather than a research study per se. For instance, we did not include a control group and goals were self-selected by participants. Relatedly, the evaluation of the project was determined by self-report scaling and goal attainment, rather than objective measures such as hospital records. Secondly, the study itself was conducted in only one geographic region (though in a relatively diverse number of settings). Our results may not be applicable to different geographic locations that have different resources available or better integrated care to treat victims of IPV. An approach like TESSA might be less effective in locations where resources are scarcer, whereas it might be redundant in other locations where health and social services are better integrated (e.g., those that provide services within the same health system). Finally, the descriptive nature of the study includes only a baseline perspective and justification of the program model, rather than an examination of program effectiveness.

These limitations notwithstanding, we found that the design of the health advocacy intervention shows promise towards filling the gaps between IPV and healthcare service delivery systems. Future studies should examine whether these self-reported goal attainment scales translate into other areas of improvement in health and wellbeing.

## Figures and Tables

**Table 1:** Theoretical influences for the Technology Enhanced Screening and Supportive Assistance (TESSA) health advocacy program.

Theoretical Influence	Description
Motivational Interviewing	Collaborative conversation style to draw out a client’s motivation and commitment to change.
Solution-Focused Brief Therapy	Goal-directed approach to draw out a client’s ideas and plans for change.
Trauma-Informed Care	Patient-centered communication approach to recognize the influence of past trauma on health behavior.

**Table 2:** Health advocacy client demographics (*n* = 419).

Characteristic	*n*	%
Gender		
Female	334	79.7%
Not-Female *	6	1.4%
Missing	79	18.9%
Age **	36.93 years	10.38 (SD)
Hispanic		
Yes	129	30.8%
No	134	32.0%
Missing	156	37.2%
Race		
White	148	35.3%
Black	87	20.8%
Bi/Multi Racial	22	5.3%
Other Race	35	8.4%
Missing	127	30.3%
Referral Organization		
IPV Organization	353	84.2%
Police Department	17	4.1%
Clinic	49	11.7%

* Includes male and gender non-conforming. Combined due to small cell sizes. ** 139 missing ages (most commonly because clients were not required to report their demographic information to receive services).

**Table 3:** Description of the goal attainment scale by the goal and number of clients selecting the baseline goals.

	−2	−1	0	+1	+2	Average
**Healthcare Management**						−0.51
Obtain insurance or other healthcare coverage	Has not explored healthcare coverage options (*n* = 29)	Has not identified healthcare coverage (*n* = 34)	Has identified eligible healthcare coverage but not yet applied (*n* = 96)	Applied for coverage but has not yet been approved (*n* = 28)	Has obtained insurance coverage (*n* = 12)	−0.20
Establish a primary care provider (PCP)	Using ER for primary care in past 90 days (*n* = 13)	No identified PCP (*n* = 48)	Identified PCP but not apt (*n* = 15)	Has future appointment with PCP (*n* = 6)	Has PCP and completed at least one appointment (*n* = 1)	−0.80
Obtain dental care	Not pursuing dental treatment (*n* = 13)	No provider identified, using alternative methods to manage dental problems (*n* = 37)	Has identified dental care provider, but does not have appointment (*n* = 14)	Has appointment scheduled for dental care (*n* = 2)	Has located dental care provider and completed at least 1 appointment (*n* = 1)	−0.88
Obtain vision care	Not pursing vision treatment (*n* = 5)	No vision provider identified, using alternative methods to manage vision problems (*n* = 19)	Has identified vision provider, but does not have appointment (*n* = 19)	Has appointment scheduled for vision care (*n* = 4)	Has located vision provider and completed at least 1 appointment (*n* = 2)	−0.43
Obtain mental/behavioral health care	Using emergency services for mental health crisis (*n* = 4)	No identified mental health provider (*n* = 28)	Identified mental health provider, but no appointment yet scheduled (*n* = 10)	Has future appointment with mental health provider (*n* = 1)	Has identified mental health provider and has completed at least one appointment (*n* = 3)	−0.63
Obtain specialty care	Not pursuing specialty care services (*n* = 1)	No identified specialty care provider (*n* = 24)	Has identified type of specialty care needed, but does not have appointment (*n* = 10)	Has appointment scheduled with specialist (*n* = 3)	Has completed at least one appointment with specialist (*n* = 4)	−0.36
Obtain well-woman exam	Has not had a well woman exam in over 2 years (*n* = 12)	Has no identified provider selected (*n* = 16)	Has identified appropriate provider but no appointment (*n* = 8)	Has appointment for well women exam scheduled (*n* = 2)	Has followed guidelines/recommendations for obtaining regular well woman exams (*n* = 0)	−1.00
Obtain prescription	Using emergency department for emergency medication needs (*n* = 2)	Has not identified prescription assistance (*n* = 19)	Identified prescription assistance but does not have medication (*n* = 9)	Has obtained prescriptions but does not have plan for ongoing care (*n* = 2)	Has prescriptions with follow up care in place (*n* = 2)	−0.5
Obtain other preventative screening	No preventative health screenings in past year (*n* = 4)	Has not identified provider or location for preventative health screening (*n* = 8)	Has identified provider/location to receive preventative health screening, but has not made appointment (*n* = 1)	Has appointment scheduled for preventative health screening (*n* = 1)	Has followed guidelines for obtaining recommended health screening (*n* = 1)	−0.87
Obtain supplies needed for health management	Using emergency department for needed. medical equipment/supplies (*n* = 0)	Has documented medical need for supplies but no resource to obtain them (*n* = 2)	Has identified resource for help in obtaining needed equipment/supplies; but not yet received them (*n* = 2)	Has obtained needed supplies but no ongoing resource (*n* = 2)	Has needed supplies and resource for obtaining ongoing supplies or equipment (*n* = 0)	0.00
**Safety**						−0.05
Address safety at home or in vehicles	Intentionally attempts to escalate conflict/risk at home (*n* = 2)	No identified methods to increase safety at home (*n* = 4)	Has identified methods to increase safety at home, but yet to implement them (*n* = 6)	Has implemented steps to increase safety at home (*n* = 6)	Has implemented steps to increase safety at home, and identifies one observable benefit (*n* = 0)	−0.11
Address safety during daily activities	Intentionally attempts to escalate conflict during daily activities (*n* = 0)	No identified methods to increase safety during daily activities (*n* = 1)	Has identified methods to increase safety during daily activities, but yet to implement them (*n* = 1)	Has implemented steps to increase safety during daily activities (*n* = 1)	Has implemented steps to increase safety during daily activities and identifies one observable benefit (*n* = 0)	0.00
Address information safety	Intentionally attempts to escalate risk of information (*n* = 0)	No identified methods to increase safety of information (*n* = 0)	Has identified methods to increase information safety, but yet to implement them (*n* = 0)	Has implemented steps to increase information safety (*n* = 1)	Has implemented steps to increase information safety and identifies one observable benefit (*n* = 0)	1.00
**Self-Care**						−0.34
Increase self-regulation	Increase in escalated body response, panic attacks (*n* = 1)	No identified methods for controlling physiological functions (*n* = 5)	Has identified new method of self-regulation but has not yet implemented it (*n* = 5)	Has adopted new method to increase self-regulation and implemented into routine (*n* = 3)	Has adopted a new method to increase self-regulation and can identify one observable benefit (*n* = 0)	−0.29
Work on personal nutrition	Actively abandoned nutrition goals (*n* = 0)	No identified nutritional goals (*n* = 4)	Has created personal nutrition goals, but not yet implemented (*n* = 5)	Has created personal nutrition goals and implemented those into routine (*n* = 0)	Actively pursuing nutrition goals and can identify one observable benefit (*n* = 0)	−0.44
Develop or enhance social support	Socially isolated, only relationships are destructive or dangerous (*n* = 1)	Has no identified support systems (*n* = 3)	Has identified potential support system but has not attempted to connect (*n* = 1)	Has identified potential supports and has made efforts to connect with them (*n* = 2)	Has identified support system that she regularly engages with for support (*n* = 0)	−0.43
Develop a strategy to increase physical activity	Significantly decreased amount of physical activity (*n* = 0)	No identified goals for increasing physical activity (*n* = 2)	Has identified ways to increase physical activity but not yet implemented (*n* = 2)	Has increased physical activity (*n* = 0)	Has increased physical activity and can identify one observable benefit (*n* = 1)	0.00
Reduce use of substances or self-medication	Engages in high risk behaviors, significant increase in use of substances (*n* = 0)	No identified healthy coping skills, slight increase in use of substances (*n* = 1)	Identified healthy coping skills, no change in use of substances (*n* = 0)	Has decreased use of substances and increased healthy coping skills (*n* = 3)	Has decreased use of substances, increased healthy coping skills, and can identify at least one observable benefit (*n* = 0)	−0.25
Create time for hobby/leisure activities	Abandoned goal of new leisure activity (*n* = 0)	No identified hobby or leisure activity (*n* = 1)	Identified new hobby or leisure activity but not yet implemented (*n* = 2)	Identified new hobby or leisure activity and implemented into routine (*n* = 0)	Has adopted new hobby/leisure activity and can identify one observable benefit (*n* = 0)	−0.33
Manage current health condition	Not managing current health condition, using emergency services for maintenance (*n* = 1)	No identified plan for health maintenance (*n* = 0)	Has created a health maintenance plan, but not yet implemented (*n* = 1)	Has implemented a health maintenance plan (*n* = 1)	Has implemented a health maintenance plan and can identify at least one observable benefit (*n* = 0)	−0.33
Establish a medication adherence plan	Uses medication in an unsafe manner, often resulting in use of emergency services (*n* = 0)	No identified plan to improve medication compliance (*n* = 1)	has identified strategies to improve medication compliance but not yet implemented (*n* = 0)	Has implemented strategies to improve medication compliance (*n* = 0)	Has been taking medications as prescribed and can identify one observable benefit (*n* = 0)	−1.00
Reduce screen time/sedentary time	Abandoned goal of reducing sedentary time (*n* = 0)	No identified alternatives to screen time/sedentary activity (*n* = 1)	Has identified alternative activities to sedentary time but not yet implemented them (*n* = 0)	Has reduced sedentary time (*n* = 0)	Has reduced sedentary time and can identify one observable benefit (*n* = 0)	−1.00
